# An individual randomised efficacy trial of autologous blood products, leukocyte and platelet-rich fibrin (L-PRF), to promote ulcer healing in leprosy in Nepal: the TABLE trial protocol

**DOI:** 10.1186/s13063-021-05392-5

**Published:** 2021-07-15

**Authors:** Indra B. Napit, Dilip Shrestha, Jon Bishop, Sopna Choudhury, Santosh Dulal, Paramjit Gill, Eleni Gkini, Holly Gwyther, Deanna A. Hagge, Karuna Neupane, Jo Sartori, Gemma Slinn, Samuel I. Watson, Richard Lilford

**Affiliations:** 1 Anandaban Hospital, The Leprosy Mission Nepal, Kathmandu, Nepal; 2 Birmingham Clinical Trials Unit, College of Medical and Dental Sciences, University of Birmingham, Edgbaston, Birmingham, B15 2TT UK; 3 Institute of Applied Health Research, College of Medical and Dental Sciences, University of Birmingham, Edgbaston, Birmingham, B15 2TT UK; 4 Division of Health Sciences, Warwick Medical School, University of Warwick, Coventry, UK

**Keywords:** Leprosy, Mycobacterium leprae, Ulcer, L-PRF, Nepal, Wound

## Abstract

**Background:**

Leprosy is curable with multidrug therapy and treatment in the early stages can prevent disability. However, local nerve damage can lead to injury and consequently recurring and disfiguring ulcers. The aim of this study is to evaluate the treatment of leprosy ulcers using an autologous blood product; leukocyte and platelet-rich fibrin (L-PRF) to promote healing.

**Methods:**

This is a single-centre study in the Anandaban Hospital, The Leprosy Mission Nepal, Kathmandu, Nepal. Consenting patients (n=130) will be individually randomised in a single-blinded, controlled trial. Participants will be 18 years of age or older, admitted to the hospital with a clean, dry and infection-free chronic foot ulcer between 2 and 20 cm^2^ in size. If the ulcer is infected, it will be treated before enrolment into the study. The intervention involves the application of leukocyte and platelet-rich fibrin (L-PRF) matrix on the ulcer beds during twice-weekly dressing changes. Controls receive usual care in the form of saline dressings only during their twice-weekly dressing changes. Primary outcomes are the rate of healing assessed using standardised photographs by observers blind to allocated treatment, and time to complete re-epithelialization. Follow-up is at 6 months from randomisation.

**Discussion:**

This research will provide valuable information on the clinical and cost-effectiveness of L-PRF in the treatment of leprosy ulcers. An additional benefit is the evaluation of the effects of treatment on quality of life for people living with leprosy ulcers. The results will improve our understanding of the scalability of this treatment across low-income countries for ulcer healing in leprosy and potentially other conditions such as diabetic ulcers.

**Trial registration:**

ClinicalTrials.govISRCTN14933421. Registered on 16 June 2020

## Administrative information

Note: the numbers in curly brackets in this protocol refer to SPIRIT checklist item numbers. The order of the items has been modified to group similar items (see http://www.equator-network.org/reporting-guidelines/spirit-2013-statement-defining-standard-protocol-items-for-clinical-trials/).

**Table:** 

Title {1}	An individual randomised efficacy trial of autologous blood products, leukocyte and platelet-rich fibrin (L-PRF), to promote ulcer healing in leprosy in Nepal: the TABLE trial protocol.
Trial registration {2a and 2b}.	ISRCTN, ISRCTN14933421. Registered on 16 June 2020. Prospectively registered.
Protocol version {3}	Version 2.1; 03.06.21
Funding {4}	National Institute for Health Research (NIHR) Research and Innovation for Global Health Transformation (RIGHT). Transforming the treatment and prevention of leprosy and Buruli ulcers in LMICs. NIHR 200132.
Author details {5a}	^1^Anandaban Hospital, The Leprosy Mission Nepal, Kathmandu, Nepal. ^2^Institute of Applied Health Research, College of Medical and Dental Sciences, University of Birmingham, Edgbaston, Birmingham, B15 2TT, UK. ^3^Division of Health Sciences, Warwick Medical School, University of Warwick, UK ^4^Birmingham Clinical Trials Unit, College of Medical and Dental Sciences, University of Birmingham, Edgbaston, Birmingham, B15 2TT, UK.
Name and contact information for the trial sponsor {5b}	University of Birmingham, Edgbaston, Birmingham, B15 2TT, UK. Delegated responsibility for clinical management of patients applies to the Anandaban Hospital, The Leprosy Mission Nepal, Kathmandu, Nepal.
Role of sponsor {5c}	The sponsor played no part in study design; collection, management, analysis, and interpretation of data; writing of the report; and the decision to submit the report for publication.

## Introduction

### Background and rationale {6a}

Leprosy is a chronic infectious disease that causes neuritis with loss of sensation. Neuropathy is caused primarily by inflammatory episodes called ‘reactions’, which can occur in 30–50% of leprosy cases in response to live *Mycobacterium leprae* or residual antigens persisting for years in skin and nerve tissues after curative treatment [1, 2]. Neuritis can develop and recur at any time before, during or even years after leprosy treatment [3]. The combination of loss of sensation and deformities leads to the presentation of ulcers, often with the first presentation within 4–5 years of leprosy diagnosis, and then an increased lifetime risk of recurrent ulcers, which can lead to deformity and permanent disability.

Nepal, with over 3200 new cases of leprosy annually, is one of 23 priority countries for the World Health Organization’s (WHO) global national leprosy programme [4]. The prevalence rate of leprosy is rising, though this may be due to improved reporting and active case finding [4]. While leprosy is curable with multidrug therapy, Nepalese people may hide early symptoms due to stigma based on cultural perceptions that leprosy is a punishment for former life transgressions [5]. Thus, there are often delays in presentation of leprosy, which can lead to the development of complex ulcers requiring long hospital stays. Furthermore, weight bearing and physical activity are often adversely associated with healing rates of plantar ulcers [6] but resting to prevent or heal an ulcer is impractical for many affected people who need to work to earn their living.

People afflicted with recurrent ulcers suffer severe consequences in terms of loss of function, loss of earnings, stigma, and often severe mental distress [7]. This protocol concerns the evaluation of a promising intervention to promote the healing of leprosy ulcers, and so improve wellbeing and quality of life for people living with leprosy. Findings would also be relevant to other ulcerative conditions, particularly diabetes, where ulcers can similarly result from peripheral nerve damage.

Leprosy ulcers heal slowly, because they are large and/or deep when they present and possibly because the skin is less moist due to damage to autonomic nerves. Slow healing leads to an extended stay in hospital, which has implications for the patients, their family members and for hospitals. Current methods of topical treatment include applications of zinc tape, wax therapy, human amniotic membrane gel, topical phenytoin, saline gel, or emerging cellular therapies such as platelet-rich plasma gel [8, 9]. However, results from intervention studies are ambiguous [8] and recent Cochrane reviews have called for higher quality research on ulcer treatment and prevention in leprosy, specifically advocating for randomised controlled trials (RCTs), ‘blinding’ of outcome measurement and appropriate sample size [8, 10].

Autologous platelet-rich plasma (PRP) is a cellular therapy with the potential to improve the healing of chronic wounds and avoid limb loss [11]. A Cochrane review suggested that it may be beneficial for diabetic patients, although the effect size was modest and the trials were not of high quality [12]. A trial of PRP to promote tendon healing did not find evidence that it was effective [13]. A ‘second generation’ of the treatment, leukocyte and platelet-rich fibrin (L-PRF), shows promise, albeit with inconclusive formal research evidence. Results from one longitudinal study in Chile [14] showed potential with a reduction in the size of wounds and closure of some chronic ulcers. However, patients acted as their own controls and so the cause of the recovery cannot be attributed to any particular aspect of the care. A trial of L-PRF in neuropathic diabetic ulcers also demonstrated positive results although the analysis was not by intention to treat [11].

Irrespective of these inconclusive or null findings, this therapy is currently used in low and middle income (LMIC) countries [14], including Anandaban Hospital, The Leprosy Mission Nepal. The use of the therapy here suggests that the treatment would be scalable if proven effective; however, reliable evidence from a randomised trial is needed to support its continued or expanded use given that it is more costly than conventional therapy.

### Aim and objectives {7}

The aim of this study is to evaluate the efficacy of L-PRF on healing rates for leprosy ulcers, time to healing and duration of hospital stay.

The objectives are as follows:

- Recruit 130 eligible adults.
- Randomise participants to intervention (application of leukocyte and platelet-rich fibrin (L-PRF) matrix on the ulcer beds) or standard care (saline dressings only).
- Monitor rate of healing based on two observations per week (cm^2^ per unit time).
- Monitor the time to complete re-epithelialization (up to a maximum of 70 days).
- Monitor participant activity using a pedometer.
- Measure health-related quality of life fortnightly using the EQ-5D 3L.
- Compare the main and secondary end-points between treatment arms.
- Monitor recurrences of treated ulcers, appearances of new ulcers and any anatomical changes in the limb at follow up (6 months from randomisation).
- Undertake an economic evaluation to estimate the impact of L-PRF on net population health in terms of daily and quality adjusted life years (DALY/QALYs).

### Trial design {8}

**Fig. 1:**
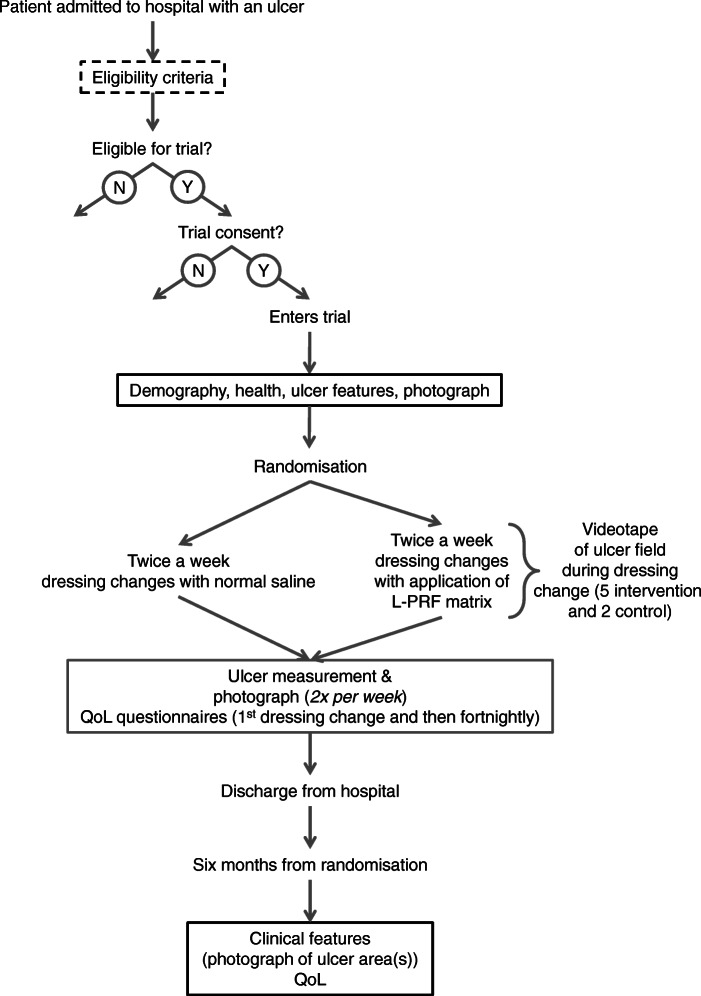
Summary of study pathway and participant timeline

A single-centre, prospective, single blinded, parallel group, 1:1 individually randomised controlled trial. Study duration is 48 months (maximum). Participants and practitioners will not be blinded, but the assessors will be completely blinded, as we describe in the section on ulcer measurements. Each participant will be followed up for up to 6 months from the point of randomisation. Consent (see ethics) and baseline data collection will precede randomisation. A summary of the study pathway is shown in Fig. 1.

## Methods: participants, interventions and outcomes

### Study setting {9}

The study will be conducted at the Anandaban Hospital currently run under The Leprosy Mission Nepal. The Anandaban Hospital lies in southern part of Lalitpur district of Nepal, approximately 20 km south of Kathmandu in the Bagmati province. Established in 1957 by The Leprosy Mission England & Wales, it is the tertiary leprosy referral hospital in Nepal, with 110 beds and provides specialist tertiary leprosy care for approximately 6000 patient visits annually from all over Nepal and the Northern part of India. It also provides general medical care for the local population.

### Eligibility criteria {10}

The inclusion criteria are as follows:

1. Patients with a chronic foot ulcer of at least 6 weeks duration due to leprosy neuropathy
2. ≥18 years of age.
3. Ulcer surface area between 2 and 20 cm^2^ inclusive.
4. Ulcer is clean, dry and free from infection.
5. Patient can provide informed consent.

The exclusion criteria are as follows:

1. Any significant medical condition, laboratory abnormality, or psychiatric illness that would prevent the participants from participating in the study (e.g. diabetes or diabetic ulcer, HIV, chronic Hep B, chronic Hep C or TB patients under active treatment).
2. Ulcer with surface area <2cm^2^ and >20cm^2^.
3. Untreated high blood pressure > 150 mm HG systolic
4. Haemoglobin less than 9 gm/dL or platelets < 100×10^3^/ul
5. Patient requires skin graft.
6. Pregnant or breast-feeding.
7. Patients with Erythema Nodosum Leprosum (ENL) or a leprosy reaction under steroid treatment.
8. Any wound that has clinical microbial infections.
9. A patient who has returned to the hospital having previously been a participant in the trial.

### Who will take informed consent {26a}

A local research fellow trained in Good Clinical Practice (GCP) will screen all admissions for eligibility. Eligible patients will be provided with a Patient Information Leaflet and verbal information as necessary about the study from a research fellow in local languages. Written informed consent will be sought the following day, once eligibility has been confirmed. Thumb or fingerprints will be requested in lieu of a signature if necessary. Translated consent forms have been back-translated according to the WHO methodology [15] for quality assurance purposes.

### Additional consent provisions for collection and use of participant data and biological specimens {26b}

Consent includes the option to give permission for the data collected to be used in future research. Participants can reject this option and still take part in the TABLE trial.

### Intervention

#### Intervention description {11a}

The intervention uses the participant’s own blood to prepare strips of leukocyte and platelet-rich fibrin matrix (L-PRF) to be applied to the ulcer bed. The process is described in detail elsewhere [16] but briefly, up to 80ml of participants’ blood will be collected twice per week at the time of dressing change. The blood will be centrifuged to obtain the fibrin matrix gel, which will be compressed and applied to the ulcer bed and then covered with a Vaseline gauge dressing. Blood collection from participants, centrifugation procedure and application of L-PRF will be done in a minor operating room following aseptic technique. All participants (including those in the control group) will be given iron and folic acid tablets. Both control and intervention groups will receive routine twice-weekly dressing changes by trained nurses or paramedics until their ulcers are healed (complete re-epithelization) up to a maximum of 70 days. Any missed dressing change sessions will be noted but not treated as a deviation from the protocol.

In the event that a participant has more than one ulcer, the largest ulcer will be selected as the index ulcer for analysis purposes before randomisation. However, all of the participant’s ulcers will receive the same treatment. Thus, if the participant is in the intervention group they will receive L-PRF treatment on all of their ulcers. Eligible patients will be offered entry in the trial at the point where their clinician judges them suitable for treatment, i.e., when the lesion is clear of any debris or infection.

#### Activity measurement

All participants will be invited to wear a pedometer (Model: Mi Smart Band 5, Model: XMSH10HM) on the ankle of their non-affected limb (or non-index case affected limb) which they will wear from the first dressing change until 42 days (the point where cross-over may occur) or discharge, whichever comes first. This will act as a proxy measure of weight bearing and enable us to monitor activity across intervention and control groups and thereby evaluate whether the level of activity is similar across groups.

#### Explanation for the choice of comparators {6b}

The comparator is usual care. Participants in the control group will receive usual care of twice-weekly standard saline dressings only and will not have blood taken. The clinical care of these participants will be identical to the intervention participants.

#### Criteria for discontinuing or modifying allocated interventions {11b}

Details of any concomitant illness or medication (present at start of the trial) will be recorded at trial entry. If any change influences the participant’s eligibility to continue in the trial, the local Principal Investigator will be informed and a decision to continue with the intervention will be made in the participant’s best interest. The intervention may also be discontinued at participant’s request. Participants who withdraw will receive usual care.

#### Strategies to improve adherence to intervention {11c}

None.

#### Relevant concomitant care permitted or prohibited during the trial {11d}

Given that weight bearing and physical activity are adversely associated with healing rates of plantar ulcers, all participants will be encouraged to rest during the trial.

#### Provisions for post-trial care {30}

Discharge information will be noted along with the participant and a family member’s contact details. Each trial participant will receive a cell phone and contact details to use in the event of any difficulties. Any readmissions at Anandaban Hospital, or any other hospitals for treatment of the same ulcer (the ‘trial ulcer’), will be recorded (both dates and duration).

#### Outcomes {12}

The main end-points will be:

1. ‘Rate of healing’ based on two observations per week of ulcer size censored at 42 days (cm^2^ per unit time). We will estimate the effect of the intervention on the change in the ulcer size per unit time, i.e. the rate of healing (see Statistical Analysis).
2. Time to complete re-epithelialization (right-censored at 42 days).

Both end-points will be analysed with and without adjustment for baseline characteristics (trial ulcer area and participants’ age), but we prefer unadjusted outcomes in randomised studies and therefore declare them as ‘primary’.

Secondary end-points:

1. Rate of healing based on two observations per week up to 70 days (cm^2^ per unit time) (calculated as described below).
2. Time to complete re-epithelisation (observed up to 70 days).
3. Generic quality of life (QoL) measured at first dressing change, fortnightly during in-patient stay and at 6-month follow-up, using the EQ-5D 3L. This scale has previously been used and validated in a Nepali population [17]. As far as we are aware, there is no valuation tariff in the Nepalese population, although this may change by the time that the analysis starts. Therefore, we propose not to prespecify the tariff but instead select the tariff at a later date. We note that tariffs are currently available for nearby countries including Sri Lanka [18] and China [19, 20].
4. Longer-term outcomes measured at 6-month follow-up from randomisation will be proportion with:
  i. Recurrence of treated ulcer;
  ii. Appearance of new ulcer;
  iii. Anatomical changes in the limb;
5. Days hospitalised prior to discharge and total (to include any readmission due to leprosy/ulcers) by 6 months.
6. Health economic information:
  i. Number of visits to any healthcare facility from discharge to the end of follow-up at 6 months.
  ii. Data on the time taken to change dressings at the twice weekly changes.

**Fig. 2:**
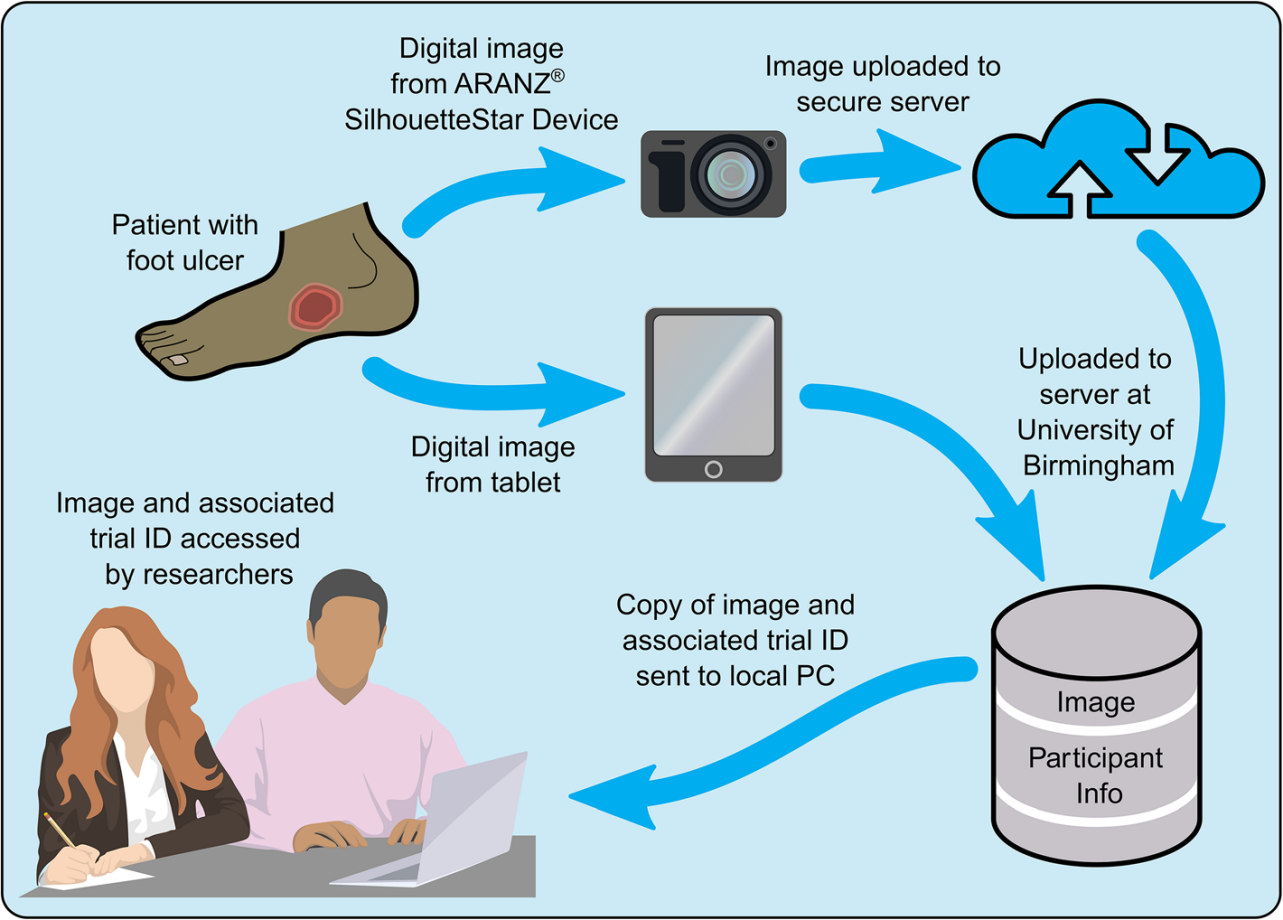
‘Closed loop’ ulcer assessment process

Rate of healing will be assessed from blindly assessed photographs (Fig. 2). However, as complete healing may trigger discharge, we will monitor for cases discharged before the blinded assessor in Birmingham has noted complete re-epithelization.

Level of activity (step count) will be collected daily and monitored across both intervention and control groups until 42 days (the point where cross-over may occur) or discharge, whichever comes first—see analysis below.

#### Participant timeline {13}

See Fig. 1 for the participant’s timeline through the trial. Patients provide consent for randomisation when they have been confirmed to be eligible (including lesion free of debris and infection). See also ‘who will take informed consent’ above.

#### Sample size {14}

Sample size is based on the two primary outcomes: rate of healing and time to complete re-epithelialization. On the latter, we assume that 70% of ulcers will heal within 42 days with standard care [21]. Further, assuming that the intervention will increase this proportion to 90% and hazards are constant and proportional, for a two-sided test of the hazard ratio with a type I error of 5% and statistical power of 80% and a 1:1 allocation ratio, 47 individuals are required in each group. To allow for withdrawals and right censoring, we aim to recruit 65 patients in each group. We expect rate of healing to provide yet more precise estimates.

#### Recruitment {15}

Eligible individuals are identified by the clinical research team and invited to participate by the local research fellow (see eligibility criteria).

### Assignment of intervention: allocation

#### Sequence generation, concealment mechanism and implementation {16a, 16b, 16c}

Participants will be enrolled sequentially and randomly allocated (1:1) to undergo L-PRF treatment or usual care using a ‘digital sealed envelope’ method [22]. An allocation table will be generated remotely by the trial statistician at The University of Birmingham to allocate participants in a 1:1 ratio at the level of the individual over the course of the trial. A random number generator will be used to generate a random sequence of the numbers between 1 to N inclusive. A permuted block randomisation method will be used by randomly selecting blocks of size 2, 4, 6 or 8 in order to maintain balance between the numbers allocated to each of the two groups. The generated table will be uploaded into the REDCap software to be used for participant enrolment. Access to the allocation table will be restricted. When a participant’s details are submitted, the trial arm and a unique study number will be assigned and revealed to the local clinician so that the randomised group that the participant is assigned to cannot be altered.

### Assignment of interventions: blinding

#### Who will be blinded {17a}

The Nepal research team, the database managers in Birmingham, the clinical staff carrying out dressing changes in the room designated for this purpose and participants themselves will be aware of participants’ randomly assigned group. Ward staff will not be informed. Researchers in Birmingham involved in the data analyses will be blinded to treatment allocation (Fig. 2).

#### Procedure for unblinding {17b}

There is no requirement for an emergency unblinding procedure.

### Data collection and management

#### Plans for assessment and collection of outcomes {18a}

**Fig. 3:**
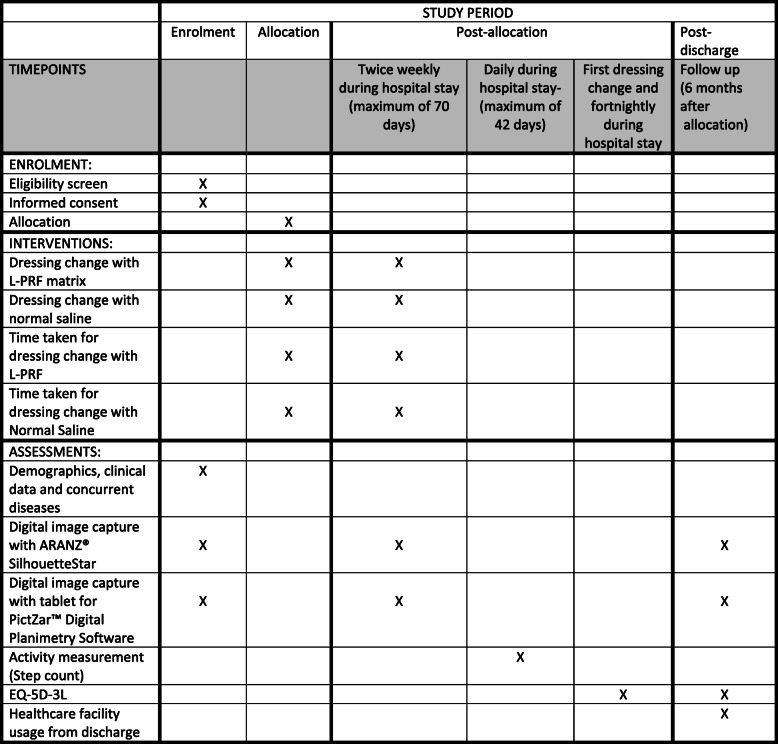
Schedule of enrolment, intervention and assessment

Demographic data (age and sex), clinical data (number and size of ulcers), concurrent diseases will be collected for all consenting participants before randomisation (Fig. 1). Data on quality of life (QoL) will be collected fortnightly from first dressing change until discharge. Ulcers will be assessed twice weekly (see below) during the participant’s hospital stay, to a maximum of 70 days, and then again at a 6-month follow-up visit. Steps taken will be collected daily until 42 days (the point where cross-over may occur) or discharge, whichever comes first. Follow-up data collection at 6 months will require an outpatient appointment or a home visit (Fig. 3). Data, including photographs of ulcers, will be collected on electronic tablets using the Research Electronic Data Capture (REDCap) system by local researchers.

### Ulcer measurements

**Fig. 4:**
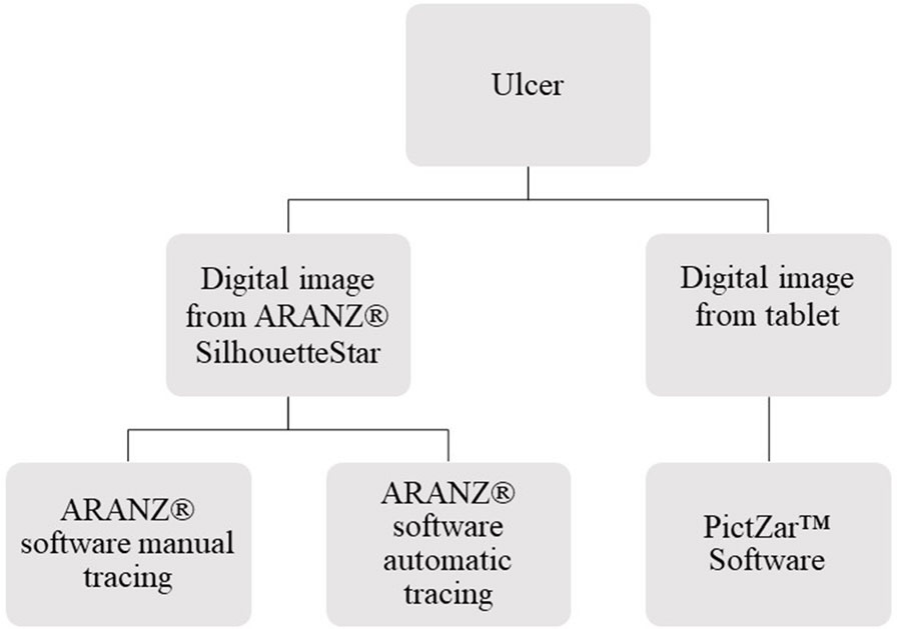
Ulcer capture and measurement process

Standardised photographs [23] will be taken twice weekly during inpatient stay dressing changes for participants in both intervention and control groups. Photographs will be obtained in Nepal using two different methods (Fig. 4). The photographs will be transferred to the University of Birmingham and the ulcer dimensions measured in three ways. Two observers will be trained to take these measurements. Both observers will be blinded to the participant’s allocated treatment. All photographs from a given participant will be assigned to the observers at random, separately for the two methods of photography. So that the measurements are not all relegated to the end of the study, they will be made in batches of ten participants reaching completion of their baseline treatments (at complete re-epithelization or 70 days from randomisation). A proportion (20%) of all ulcer photographs will be measured by both observers to test inter-rater reliability. These photographs will be selected at random.

The date at which complete re-epithelization took place will be determined by the local clinician. Photographs will be taken at the point of complete re-epithelialization and during the follow-up visit.

### ARANZ® SilhouetteStar photography

For the first two measurement methods, a photograph will be taken using the ARANZ® SilhouetteStar device (camera) and the image synchronised to the participant’s trial number and saved to the secure ARANZ® SilhouetteCentral SQL server. This will be assessed by a designated observer at the University of Birmingham. The validated ARANZ 3D wound measurement software tool will then be used to measure the ulcer in two ways. The first measurement will be made using a manual tracing method whereby the ulcer boundary will be ‘hand’ drawn in the ARANZ® software. The software will then calculate the ulcer dimensions based on this outline. The second measurement will utilise an automatic tracing feature in the software. In this instance, the observer will delineate a region of interest (extending beyond the boundary of the ulcer) and the software will automatically locate the boundary and calculate the wound dimensions.

### Digital tablet

The final measurement will use a photograph taken using the in-built camera in the tablet devices (Samsung Galaxy Tab S6). The photograph will be taken perpendicular to the ulcer. For calibration purposes, a 3-cm size clean paper ruler with date and participant’s trial identification number will be placed in the photograph frame above or below the ulcer but at the level of the skin. The photograph will uploaded to the server at the University of Birmingham and evaluated digitally by a designated observer in Birmingham using the PictZar™ Digital Planimetry Software [24] with an electronic PUSH Tool (National Pressure Injury Advisory Panel (NPIAP) at https://npiap.com/page/PUSHTool). The observer will delineate an area of interest by manually ‘painting’ the ulcer area with colour using a computer mouse. The software will then calculate the ulcer dimensions based on this profile.

All observations will be made blind to treatment allocation. The ‘closed loop’ ulcer assessment process is shown in Fig. 2. The changes in surface area can then be determined between each observation using three methods of assessment (Fig. 4).

#### Plans to promote participant retention and complete follow-up {18b}

Each trial participant will receive a cell phone to enable the research team to contact them regarding their 6-month follow up.

#### Data management {19}

Each participant will be allocated a unique participant identification number which will be used on all electronic documents and photographs. Data will be collected by researchers in Nepal and entered on to the REDCap platform hosted on a secure server at the University of Birmingham. Ulcer photographs taken using the ARANZ® device will be saved to the secure SilhouetteCentral SQL server. Data will be encrypted and access will be password restricted.

#### Confidentiality {27}

All collected data will remain confidential. All data will be stored in accordance with GCP and General Data Protection Regulations 2018 (GDPR), and the Data Protection Act 2018.

#### Plans for collection, laboratory evaluation, and storage of biological specimens for genetic or molecular analysis {33}

None

### Analyses and inference

#### Statistical methods for main endpoints {20a}

##### Time to healing

Time to healing will be analysed using a Cox proportional hazards model with and without adjustment for baseline characteristics (trial ulcer area and participants’ age) allowing for right-censoring. For the rate of healing analysis, we will define the outcome as the ulcer size in cm^2^ at each time point and include in the model time since admission, treatment status and their interaction. Our parameter of interest will be that of the interaction term, which will be interpreted as the mean difference in rate of healing (in cm^2^ per unit time) between intervention and control groups. We will analyse this model using a linear mixed-effects model with participant-level random effects and both with and without adjustment for participant characteristics. Given there are multiple primary outcomes (two outcomes, with and without adjustment, for three types of ulcer assessment), we will adjust reported p values for multiple testing using a stepdown method, which provides an efficient means of controlling the family-wise error rate [25]. We will derive the approximate distributions of the test statistics to perform the stepdown procedure using a permutation test approach, by simulating 10,000 re-randomisations of the individuals [26].

##### Measurements

The above analyses of healing rate and time of complete re-epithelialisation will be made separately for each method of measurement. Photographs will be assessed blind to allocated treatment by two trained observers at the University of Birmingham (Fig. 2). Inter-rater reliability of the measurements will be made on 20% of ulcer photographs and assessed using interclass correlation coefficients.

Measurements are based on the ARANZ ® and tablet-based cameras (see Fig. 2). The ARANZ® images are measured in two ways: (1) manual tracings and (2) automatic tracings of wounds using a camera specifically designed to obtain standardised photographs of ulcers for scientific purposes. The distance of the camera from the ulcer surface is controlled by triangulation of three laser lights. The images taken by the camera in the tablet are calibrated digitally by reference to a measurement ruler in the frame. The areas (cm^2^) are then generated within the different software packages. We will measure, agreement between the methods of measurement:

1. The Push tool vs. the automated ARANZ® method
2. The automated vs. manual ARANZ® method

#### Interim analyses {21b}

An interim analysis will be conducted when at least 49 participants have been followed-up for a minimum of 42 days. The rationale for this analysis is the detection of a ‘penicillin-like’ benefit or statistically significant negative effect of the treatment on either of the two main endpoints. A statistical threshold of 0.01, one-sided (0.02 two-sided) will be used for either of the two main endpoints. In the event that more than 10% of control participants cross-over from one arm to another at 42 days, we will consider performing a complier average causal effects analysis to the 70 day outcome.

#### Missing data {20c}

We will analyse by intention to treat. In the (extremely unlikely) event that some participants withdraw before complete healing and that rate differs between groups, a sensitivity analysis will be applied. We will explore the patterns and extent of any missing data, particularly those relating to the two main endpoints. We do not plan to impute missing values, but may consider the use of multiple imputation or other strategies within the sensitivity analysis if necessary.

#### Methods for additional analyses {20b}

##### Quality of life

Quality of life (QoL) will be analysed by calculating and comparing area under the curve (AUC) across intervention and controls. Baseline results will be triangulated with clinical observations to avoid bias and to determine how differences in healing rates correspond to differences in quality of life end-points.

##### Activity measurement

We will also compare average daily step count between treatment and control groups as a simple difference in means (t test). Since one group may stay longer in hospital than the other and since there may be an interaction between rate of healing and step count, we will compare step counts over periods pre-set at 7, 14 and 42 days.

##### Economic evaluation

An economic evaluation of L-PRF will be undertaken. The model will estimate the impact of L-PRF on net population health in terms of daily and quality adjusted life years (QALYs/DALY) over a long-term time horizon. The effectiveness of the treatment will be reflected by estimating the statistical relationship between the primary end-point in the efficacy trial and HRQoL (health-related quality of life) based on participants’ completion of the generic quality of life instruments. This analysis will also reflect other characteristics of participants that are potentially prognostic and predictive of the efficacy of L-PRF. The analysis will provide a careful assessment of how the cost-effectiveness of L-PRF varies according to its local acquisition cost, as well as other parameters that could vary between localities. Full uncertainty analysis will be undertaken to establish whether there is sufficient evidence to support local funding of L-PRF at a given acquisition cost or whether additional evidence generation is necessary and worthwhile. We shall develop the model before the data to populate it are available and our work-plan will included a review of the relevant literature in countries with both high and low incomes.

##### Reporting

Where a participant withdraws from the intervention as a whole, no further data will be collected. Where the participant withdraws from the randomised intervention but agrees to contribute data, they will be followed-up to the point of discharge as an inpatient. The trial will be reported in line with the CONSORT (Consolidated Standards of Reporting Trials) Standards [27, 28].

#### Plans to give access to the full protocol, participant level data and statistical code {31c}

The full protocol, non-identifiable participant level data and statistical code may be available for sharing once the trial has ended. All requests will be approved by the Chief Investigator (CI), Professor Richard Lilford (r.j.lilford@bham.ac.uk).

### Oversight and monitoring

#### Composition of the co-ordinating centre and trial steering committee {5d}

The trial will be overseen by the Trial Management Group (TMG) and a Trial Steering Committee (TSC). The TMG will be responsible for the day-to-day management of the trial. It includes individuals at the University of Birmingham (Chief Investigator, Trial Manager, Trial Co-ordinator, Clinical Trials Unit management staff) and Anandaban Hospital, The Leprosy Mission Nepal (Principal Investigator, local Project Manager and patient representatives). The TMG will meet monthly by teleconference. The Trial Steering Committee provides overall supervision of the trial and will ensure that it is conducted in accordance with the principles of Good Clinical Practice and other relevant regulations. Meetings will be scheduled before enrolment and after each meeting of the Independent Data Monitoring Committee and more frequently during the analysis phase. The TSC includes an independent chair and members with clinical expertise.

#### Composition of the Data Monitoring Committee, its role and reporting structure {21a}

The Data Monitoring Committee will review safety and efficacy data during the active phase of the trial. They will advise on the continued recruitment of trial participants. They will meet either by teleconference or face-to-face. This committee consists of an independent chair, a statistician and members with clinical and methodological expertise.

#### Adverse event reporting and harms {22}

The Principal Investigator in Nepal, Dr. Indra Napit, is responsible for recording all adverse events (AEs) and reporting any serious adverse events (SAEs) to the Chief Investigator and University of Birmingham Clinical Trials Unit (BCTU) within 24 h of becoming aware of such an event. A SAE form will be available on the data collection tablets and a database of any events will be maintained. The Trial Management Group, Chief Investigator and the BCTU will review any SAE forms. The Trial Steering Committee will periodically review all safety data and liaise with the Independent Data Monitoring Committee regarding any safety issues. Any deaths will be reported to the Sponsor irrespective of whether the death is related to the disease progression, the intervention or an unrelated event.

#### Frequency and plans for auditing trial conduct {23}

The trial is audited and monitored by the Sponsor, the University of Birmingham.

#### Plans for communicating important protocol amendments to relevant parties (e.g. trial participants, ethical committees) {25}

Any protocol amendment will be reported to the Trial Management Committee for approval. The Sponsor, University of Birmingham Biomedical and Scientific Research Ethics Committee and the Nepal Health and Research Council will subsequently be notified.

#### Dissemination plans {31a}

We will publish and disseminate through the usual academic channels, including peer reviewed journals and at academic conferences. Our dissemination plans include close liaison with The Leprosy Mission to engage affected people and communities and to ensure that the results are disseminated widely. Other dissemination plans include bite-sized research reports in lay format, public announcements in communities in LMICs, policy briefings, print and online media, the Chief Investigator’s News Blog (680+ subscribers), institutional and professional social media accounts and websites.

## Discussion

This protocol describes an individually randomised control trial to evaluate a treatment using autologous blood products L-PRF, to promote ulcer healing in leprosy. Despite the promising anecdotal evidence regarding this treatment, high-quality, empirical data are currently unavailable [12, 14].

Most of the evidence on ulcer healing, including the Cochrane review [12], is based on proportion of ulcers healed (completely epithelialized) by 42 days. This is sub-optimal because complete epithelialisation is a clinical decision that is also used to decide on discharge. This provides an opportunity for observer (outcome) bias. For this reason, we will measure rate of healing. These measures are all made centrally (in Birmingham) by two observers who are ‘blind’ to intervention status.

Activity measurements are not a primary outcome measure in our study. However, by collecting step-count daily and over the course of the inpatient stay over the periods specified above, we can exclude activity as a confounding factor in any differences found in ulcer healing rates between intervention and control groups.

We are also planning to do a range of nested studies. Given sufficient time and resources, we hope to video tape a subset of dressing changes (five intervention and two controls selected at random in Birmingham) as a means of photograph supervision and for additional quality control. We will also develop a more detailed health economics protocol. Finally, we will develop follow on studies based on the results of the trial.

We are using three methods to measure the rate of ulcer healing and time to complete re-epithelialisation. Readers might wonder at such an extensive set of methods and consider this ‘overkill’. Our rationale for using these multiple ulcer measurement methods is two-fold. First, it is crucially important in our judgement to create a situation where results cannot be explained away by measurement error (for all that measurement error is more of a threat to precision than to accuracy). Second, we wish to contribute to the methodological literature on ulcer measurement. As investigators we hope that the results will be consistently null or positive (or even negative). However, we realise that we may end up with results that toggle around the conventional statistical threshold , say, according to ulcer measurement method or whether or not adjustment had been carried out for any discrepancy in ulcer size at base-line. In that case, we will simply report the different results and allow the reader to make an interpretation. We will not (miss) use statistical tests as decision rules.

By using a randomised control method, alongside standardised blindly assessed photographs, and information on activity, this research will provide robust evidence on the clinical and cost-effectiveness of L-PRF in the treatment of leprosy ulcers. Data collection and ulcer assessment follows a ‘closed-loop’ such that the photograph and image can follow only one pathway from camera to completed measurement. An additional benefit is the evaluation of the effects of treatment on quality of life for people living with the effects of leprosy. The findings will also be relevant to other ulcerative conditions, particularly diabetes, where ulcers similarly result from peripheral nerve damage. The results will improve our understanding of the scalability of this treatment across low-income countries for ulcer healing in leprosy and other similar conditions.

### Trial status

Protocol version number: Version 2.0; 25.11.20

The TABLE trial is currently recruiting. The first patient was randomised on 18th September 2020. The Trial Management Group (TMG) and a Trial Steering Committee (TSC) met before the first patient was enrolled. We anticipate that it will take approximately 12–18 months to complete the recruitment phase.

## Abbreviations

AUC: Area under the curve
BSREC: University of Birmingham Biomedical and Scientific Research Ethics Committee
CONSORT: Consolidated Standards of Reporting Trials
DALY: Disability adjusted life year
ENL: Erythema Nodosum Leprosum
EQ-5D 3L: 5-Dimensional European Quality of Life Questionnaire
HIV: Human immunodeficiency viruses
HRQoL: Health-related quality of life
L-PRF: Leukocyte and platelet-rich fibrin
LMIC: Low- and middle-income countries
NHRC: Nepal Health Research Council
NIHR: National Institute for Health Research
NPIAP: National Pressure Injury Advisory Panel
PRP: Platelet-rich plasma
PUSH: Pressure Ulcer Scale for Healing
QALY: Quality adjusted life year
QoL: Quality of life
RCT: Randomised controlled trials
REDCap: Research Electronic Data Capture
RIGHT: Research and Innovation for Global Health Transformation (RIGHT)
TABLE: Trial of Autologous Blood products to promote ulcer healing in LEprosy
TMG: Trial Management Group
TSC: Trial Steering Committee
TB: Tuberculosis
TLMN: The Leprosy Mission Nepal
W-CAHRD: Warwick Centre for Applied Health Research & Delivery
WHO: World Health Organization
